# Analysis of the Active Constituents and Evaluation of the Biological Effects of *Quercus acuta* Thunb. (Fagaceae) Extracts

**DOI:** 10.3390/molecules23071772

**Published:** 2018-07-19

**Authors:** Mi-Hyeon Kim, Dae-Hun Park, Min-Suk Bae, Seung-Hui Song, Hyung-Ju Seo, Dong-Gyun Han, Deuk-Sil Oh, Sung-Tae Jung, Young-Chang Cho, Kyung-Mok Park, Chun-Sik Bae, In-Soo Yoon, Seung-Sik Cho

**Affiliations:** 1 Department of Pharmacy, College of Pharmacy and Natural Medicine Research Institute, Mokpo National University, Muan, Jeonnam 58554, Korea; mee4523@naver.com (M.-H.K.); tmdgml7898@naver.com (S.-H.S.); 2 Department of Nursing, Dongshin University, Naju, Jeonnam 58245, Korea; dhj1221@hanmail.net; 3 Department of Environmental Engineering, Mokpo National University, Muan, Jeonnam 58554, Korea; minsbae@hotmail.com; 4 Department of Manufacturing Pharmacy, College of Pharmacy, Pusan National University, Geumjeong, Busan 46241, Korea; hlhl103@naver.com (H.-J.S.); hann009584@gmail.com (D.-G.H.); 5 Jeonnam Forest Resurce research Institue, Naju, Jeonnam 58213, Korea; ohye@korea.kr; 6 Jeollanamdo Wando Arboretum, Wando, Jeonnam 59105, Korea; jungtai7167@korea.kr; 7 Department of Pharmacy, College of Pharmacy, Chonnam National University, Gwangju 61186, Korea; yccho@jnu.ac.kr; 8 Department of Pharmaceutical Engineering, Dongshin University, Naju, Jeonnam 58245, Korea; parkkm@dsu.ac.kr; 9 College of Veterinary Medicine, Chonnam National University, Gwangju 61186, Korea; csbae210@chonnam.ac.kr

**Keywords:** *Quercus acuta* leaf, antioxidant, antibacterial activity, *Staphylococcus aureus*

## Abstract

We evaluated the antioxidant and antibacterial activity of hexnane, ethyl acetate, acetone, methanol, ethanol, and water extracts of the *Quercus acuta* leaf. The antioxidant properties were evaluated by 1,1-diphenyl-2-picrylhydrazyl (DPPH) free radical scavenging activity, reducing power, and total phenolic content. Antibacterial activity was assessed against general infectious pathogens, including antibiotic-resistant clinical isolates. The methanolic extract showed the highest DPPH radical scavenging activity and total phenolic content, while the reducing power was the highest in the water extract. The ethyl acetate extract showed the best antibacterial activity against methicillin-resistant *Staphylococcus aureus* (MRSA) strains. Additionally, it displayed antibacterial activity against *Staphylococcus aureus* KCTC1928, *Micrococcus luteus* ATCC 9341, *Salmonella typhimurium* KCTC 1925, *Escherichia coli* KCTC 1923, and eight MRSA strains. These results present basic information for the possible uses of the ethanolic and ethyl acetate extracts from *Q. acuta* leaf in the treatment of diseases that are caused by oxidative imbalance and antibiotic-resistant bacterial infections. Six active compounds, including vitamin E, which are known to possess antioxidant and antibacterial activity, were identified from the extracts. To the best of our knowledge, this is the first study that reports the chemical profiling and antibacterial effects of the various QA leaf extracts, suggesting their potential use in food therapy or alternative medicine.

## 1. Introduction

*Quercus acuta* (QA) is widely distributed in the southern part of Korea, China, Japan, and Taiwan [1]. It is mainly cultivated as an ornamental plant in Japan, and its fruit (acorn) is the main ingredient in acorn jelly, which is a popular traditional food in Korea [2]. Till date, only a few studies have investigated the pharmacological activity of various QA extracts and its active constituents. The QA trunk extract and its two constituents, 4,5-di-*O*-galloyl (+)-protoquercitol and 3,5-di-*O*-galloyl protoquercitol, have been reported to possess antibacterial effect against both gram positive and gram-negative bacteria [3]. Moreover, (+)-catechin, (-)-epicatechin, taxifolin, taxifolin 3-*O*-β-d-glucopyranoside, taxifolin 4′-*O*-β-d-glucopyranoside, procyanidin B-3, and (+)-lyoniresinol 3α-*O*-β-d-xylopyranoside, which are antioxidant phytochemicals [4,5], have been isolated from the stems of QA [6]. Recently, we reported the potent xanthine oxidase inhibitory and antihyperuricemic activities of the ethylacetate extract of QA leaf and its twelve active constituents [1].

However, in our previous study, optimization of the extraction conditions with respect to various solvents and marker compounds was not conducted. The above-mentioned literature reporting the pharmacological effects of the various QA extracts can lead us to expect further development of pharmaceuticals and functional foods containing QA extracts in the future, but no positive results have yet been reported. To facilitate the pharmaceutical and food industrialization of the QA extracts, additional chemical profiling and optimization data is the need of the hour. Moreover, to the best of our knowledge, there have been no studies on the antibacterial effects of QA leaf extracts, which warrants further investigation.

In the present study, we prepared various extracts of the QA leaf using hexane, ethyl acetate, acetone, ethanol, methanol, and water in order to determine the optimal extraction conditions with respect to biological activity and phytochemical profiles. Gas chromatography-mass spectrometry (GC-MS) and high-performance liquid chromatography (HPLC) were used for the chemical profiling of the extracts prepared with the various solvents. Next, the antioxidant and antibacterial activities of the optimized QA leaf extracts were examined. The antioxidant activity was confirmed by measuring 1,1-diphenyl-2-picrylhydrazyl (DPPH) radical scavenging activity, reducing power, and the total pheolic content. On the other hand, the antibacterial activity was confirmed using the minimum inhibitory concentration (MIC) test against general infectious bacteria and antibiotic-resistant strains of clinical origin.

## 2. Results and Discussion

### 2.1. Analysis of Active Substances

**Figure 1:**
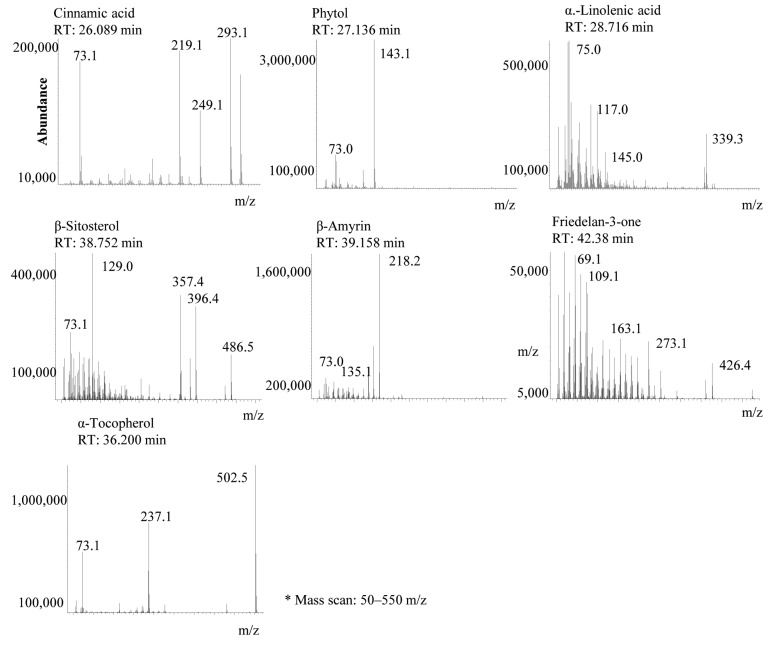
Representative gas chromatography-mass spectrometry (GC-MS) chromatogram to show the bioactive constituent profiles of QA (*m/z*: mass-to-charge ratio).

In the present study, we identified the active substances in the QA leaf extracts using the GC-MS and HPLC systems. The analytical conditions for the GC-MS and HPLC methods were the same as those previously reported [1]. The active constituents were identified as cinnamic acid, phytol, α-linolenic acid, α-tocopherol, β-sitosterol, β-amyrin, and friedelin-3-ol from the hexane, ethyl acetate, and acetone extracts. Total ion current (TIC) data from the GC-MS chromatogram are shown in Figure 1. In our previous study, we identified α-linolenic acid and α-tocopherol from the leaves of QA [1]. In the ethanol, methanol and water extracts, cinnamic acid, phytol, α-linolenic acid, and α-tocopherol were not identified. Cinnamic acid, phytol, α-tocopherol, β-sitosterol, and β-amyrin have been reported as sources of antioxidant activity. Cinnamic acid, phytol, β-sitosterol, and friedelin-3-ol have also been reported to have antibacterial activities.

The results of the comparison of the seven active ingredient contents for each organic solvent extract are as follows: hexane ex (35.28%) > ethyl acetate ex (35.2%) > acetone ex (29.7%) > ethanol (26.07%) > methanol ex (0.79%) > water ex (not detected). The use of a nonpolar organic solvent increased the extraction rate of the seven components (Table 1). A previous study reported that cinnamic acid showed antioxidant activity including free radical scavenging properties [7] and showed antibacterial activity against most Gram-negative and Gram-positive bacteria, with MIC values that were higher than 5 mM. Additionally, cinnamic acid was found to exhibit antibacterial activity against Mycobacterium tuberculosis [8]. α-tocopherol is a well-known antioxidant and antibacterial compound. Gulcin et al. reported that α-tocopherol showed reducing power, superoxide anion radical scavenging activity, metal chelating ability, hydrogen peroxide scavenging activity, and inhibition of lipid peroxidation [9]. Phytol is an acyclic diterpene alcohol that has antioxidant and antibacterial activity. Santos et al. reported that phytol removes hydroxyl radicals and nitric oxide and it also prevents the formation of thiobarbituric acid reactive substances [10]. The antibacterial mechanism of phytol is not fully established. It is also suggested that protein and enzyme inactivation are representative of the inhibition of microbial growth [11]. Ghaneian et al. documented that phytol showed antibacterial activity against *Escherichia coli* (*E coli*), *Candida albicans*, and *Aspergillus niger*, and the MIC (minimum inhibitory concentration) was 62.5 μg/mL. However, *Staphylococcus aureus* was resistant to phytol [12]. β-sitosterol, which is a typical sterol molecule, is known to have moderate antioxidant and antibacterial properties. It exerts positive effects in vitro by decreasing the levels of reactive oxygen species. β-sitosterol is reported to decrease levels of liver lipid peroxides and exhibited a protective action against *1*,2-dimethylhydrazine-induced depletion of antioxidants like catalase, superoxide dismutase, and glutathione peroxidase in colonic and hepatic tissues from animals [13]. β-Sitosterol has also been reported to have antibacterial activity against *E. coli*, *Pseudomonas aeruginosa*, *Staphylococcus aureus* (*S. aureus*), and *Klebsiella pneumoniae* [14]. Sunil et al. reported that β-amyrin showed very good IC50 values in DPPH (IC_50_ = 89.63 ± 1.31 μg/mL), hydroxyl (IC_50_ = 76.41 ± 1.65 μg/mL), nitric oxide (IC_50_ = 87.03 ± 0.85 μg/mL), and superoxide (IC_50_ = 81.28 ± 1.79 μg/mL) radical scavenging effects. Moreover, β-amyrin showed high reducing power and suppressed lipid peroxidation [15]. Odeh et al. purified friedelin-3-ol from *Pterocarpus santalinoides* and evaluated its antibacterial activity; friedelin-3-ol had a MIC value of 10 μg/mL for MRSA, *Helicobacter pylori* (*H. pylori*), and *E. coli*, and the minimum bactericidal/fungicidal concentration (MBC/MFC) values against MRSA, *H. pylori*, *Candida krusei*, *S. aureus*, *Streptococcus pneumoniae*, and *Candida tropicalis* ranged from 10 μg/mL to 40 μg/mL [16].

**Figure 2:**
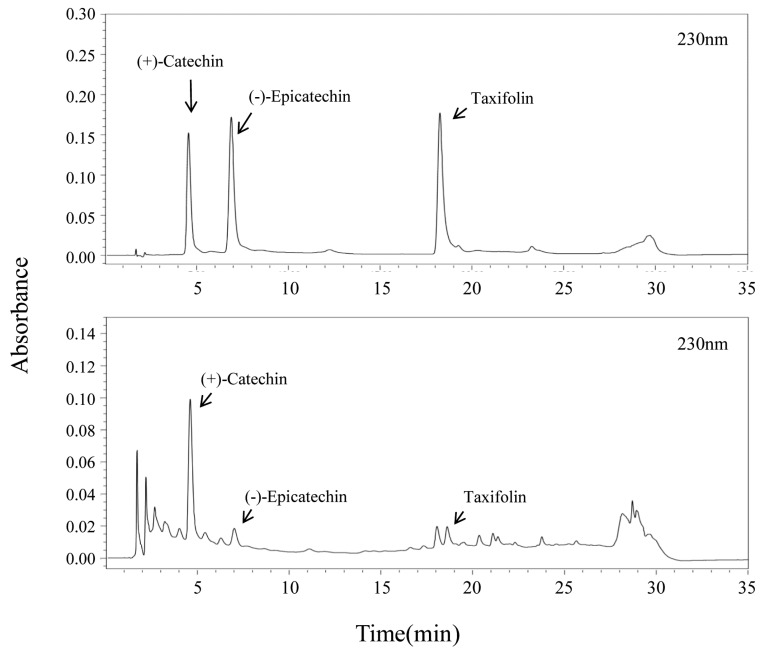
Chromatogram of standard and QA leaf extract.

The active constituents identified and the representative chromatograms of the standard mixture and sample extracts are shown in Figure 2. The main peak was identified as (+)-catechin in the chromatographic profiles. Additionally, two minor compounds, (-)-epicatechin and taxifolin, were also identified. Oh et al. had previously reported that QA contains flavans and flavonols, such as catechins and taxifolin, which is in agreement with our results [6]. We compared the content of these three active compounds in the various extracts. The extraction yield and content of the three compounds were the highest in the methanol extract (Table 2). The content of (+)-catechin, (-)-epicatechin, and taxifolin in the methanol extract was 27 mg/g, 3 mg/g, and 2.6 mg/g. The total amount of the three components was 32.6 mg/g, which was the highest in the methanol extract. Therefore, the methanol extract was a flavonol-/flavan-3-ol rich extract.

### 2.2. Antioxidant Activity and Total Phenolic Content of the QA Extracts

The antioxidant activity of the QA extracts was evaluated using DPPH free radical scavenging and reducing power assays. Additionally, the total phenolic content (mg/g as gallic acid) was also measured. This is because phenolic compounds are widely known to contribute to the recovery of various diseases that are caused by an imbalance of oxidative stress or infection. The DPPH radical scavenging activity is shown in Table 3. The methanol extract showed the highest DPPH radical scavenging activity with a half-maximal inhibitory concentration (IC_50_) of 49.58 μg/mL.

Furthermore, we evaluated the reducing power of the QA extracts. In the present study, we tested the reductive capability of the extracts by measuring the reduction of Fe^3+^. The hot water extract showed the highest activity among all of the extracts (Table 4). The reductive activity of the water extract was expressed as 171.57 ± 0.93 μg/100 μg equivalent to ascorbic acid. The total phenolic content was determined using the Folin-Ciocalteu method [17], and it was reported as gallic acid equivalents, as shown in Table 4. The phenolic content of the ethanolic extract was higher than that of the other extracts (85.2 ± 0.89 mg/g as gallic acid equivalents). The total phenolic content of the ethanolic extract was similar to that of the methanolic extract (83.25 ± 2.39 mg/g, as gallic acid equivalents). Taken together, these results indicate that the DPPH radical scavenging activity and the phenolic content were the highest in the methanol extract, while the reducing power was highest in the water extract.

As mentioned earlier, the total content of the three active flavonoids, i.e. (+)-catechin, (-)-epicatechin, and taxifolin, was 32.6 mg/g (Table 2), accounting for 39.2% of the total phenolic content in the methanol extract. Therefore, flavonoids such as (+)-catechin, (-)-epicatechin, and taxifolin are considered to considerably contribute to the antioxidant activity of QA.

### 2.3. Antibacterial Activity of QA Extracts 

Samples were subjected to extraction with different solvents in order to select the best extraction solvent conditions: hexane, ethyl acetate, acetone, methanol, ethanol, and water. First, we analyzed the inhibition effects of the QA leaf extracts on methicillin-resistant *S. aureus* 693E (MRSA 693E) through the disk diffusion method [17]. We observed that the hexane, ethyl acetate, and acetone extracts displayed antibacterial activity. Among these, the ethyl acetate extract showed the highest antibacterial activity (data not shown). In Table 5, vancomycin was used as the control; this is because vancomycin is well-known commercial antibiotic, which is used against infectious bacteria, including antibiotic-resistant strains. In Section 2.1., we described the identification of antibacterial substances such as cinnamic acid, phytol, β-sitosterol, and friedelin-3-ol from the QA leaf. The total amount of cinnamic acid, phytol, β-sitosterol, and friedelin-3-ol in each extract was calculated. The results of the comparison of the content of the four active ingredients in each organic solvent extract are as follows. Ethanol ex (19.55%) > ethyl acetate ex (14.57%) > hexane ex (14.08%) > acetone ex (13.05%) > methanol ex (0.79%) > water ex (not detected). Besides, the ethanolic extract showed the highest content of antibacterial substances, such as β-sitosterol and friedelin-3-ol, but the antibacterial activity was observed to be the highest in the ethyl acetate extract. Thus, although the ethanolic extract contains only β-sitosterol and friedelin-3-ol, these substances do not have a significant effect on antibacterial activity.

In the present study, we evaluated the potential activities of ethyl acetate extract against Gram-positive, Gram-negative bacteria, and hospital-acquired antibiotic-resistant strains, such as methicillin-resistant *S. aureus* (MRSA), vancomycin-resistant enterococci (VRE), carbapenemase producing *P. aeruginosa* (IMP), and extended spectrum β-lactamase producing *E. coli* (ESBL). This study is significant because it is the first report of the antibacterial susceptibility of the QA extract against the recently isolated MDR strain. As shown in Table 5, the ethyl acetate extract was found to have antibacterial activity against *Staphylococcus aureus* KCTC1928, *Micrococcus luteus* ATCC 9341, *Salmonella typhimrium* KCTC 1925, *E. coli* KCTC 1923, and eight MRSA strains with MIC values that were ranging from 125 to 500 μg/mL. In the present study, the ethyl acetate extract showed antibacterial activity against *S. aureus* and MRSA.

*S. aureus* commonly causes skin diseases such as atopic dermatitis. About 90% of patients with atopic dermatitis are colonized by *S. aureus* in lesional skin, whereas most healthy individuals do not harbor the pathogen [18]. *S. aureus* is often found in burn wounds and implanted deep-vein catheters, which often leads to refractory infections, or even biofilm-related sepsis. Yin et al. found that burn serum increases *S. aureus* biofilm formation via elevated oxidative stress. Importantly, antioxidants can suppress the biofilm formation and bacterial cell aggregation that is caused by burn serum [19]. These findings are closely related to our results. The antioxidant and antibacterial effects of the ethyl acetate extract are due to its broad antibacterial activity on *S. aureus* strains, including MRSA of clinical origin. Therefore, the QA extract can be expected to mitigate the oxidative imbalance that is caused by staphylococcal infection and inhibit bacterial growth.

## 3. Experimental Section

### 3.1. Plant Material and Extract Preparation

QA leaves were supplied from the Wando Arboretum (Wando, Korea). A voucher specimen (MNUCSS-QA-02) was deposited at the Mokpo National University (Muan, Korea). Air-dried and powdered QA leaves (20 g) were subjected to extraction twice with hexane, ethyl acetate, acetone, ethanol, and methanol (100 mL) at room temperature for 48 h or subjected to extraction with hot water (100 °C) for 4 h. The resultant solution was evaporated, dried, and stored at −20 °C for further experiments.

### 3.2. Chromatographic Conditions 

For the organic marker speciation, the samples were extracted individually in methylene chloride (DCM) for GC-MS analysis. The final volume for each sample was adjusted to 500 µL using a nitrogen blowdown equipment. Each aliquot was silylated prior to analysis using N,O-bis (trimethylsilyl) trifluoroacetamide (CAS# 25561-30-2) to derivatize the constituents to their trimethylsilyl-derivatives. To analyze the QA extracts, the silylated aliquot was analyzed using gas chromatography-electron impact-mass spectrometry (GC-EI-MS) with an HP-5MS capillary column (150 mm × 4.6 mm, Agilent, Santa Clara, CA, USA). The oven temperature was controlled as isothermal at 65 °C to 300 °C. All of the scanned mass spectra (50–550 amu) were examined and confirmed using the NIST 2017 mass library (Scientific Instrument Services, Ringoes, NJ, USA) [20]. The HPLC method that was developed in this study was used to quantitatively determine the (+)-catechin, (-)-epicatechin, and taxifolin content in the extracts of the QA leaves (Table 6).

### 3.3. DPPH Free Radical Assay

Sample solutions (0.5 mL) were mixed with the DPPH solution (0.4 mM, 0.5 mL) for 10 min and optical density was observed at 517 nm using a microplate reader (Perkin Elmer, Waltham, MA, USA). The radical scavenging activity was calculated as a percentage while using the following equation and the IC_50_ (μg/mL) values were also calculated [17].

DPPH radical scavenging activity (%) = [1 − (A_sample_/A_blank_)] × 100

### 3.4. Reducing Power Assay

The reducing power of the extract was determined using a previously reported method with slight modifications [17]. The extract (0.1 mL), sodium phosphate buffer (0.2 M, 0.5 mL), and potassium ferricyanide (1% *w*/*v*, 0.5 mL) were mixed and incubated at 50 °C for 20 min. After stopping the reaction with trichloroacetic acid solution (10% *w*/*v*, 0.5 mL), the mixture was centrifuged at 2000× *g* for 10 min. The supernatant was then mixed with distilled water (0.5 mL) and iron (III) chloride solution (0.1% *w*/*v*, 0.1 mL). The absorbance of the resultan mixture was measured at 700 nm, and the reducing power of the sample was expressed as ascorbic acid equivalents.

### 3.5. Total Phenolic Content

The Folin-Ciocalteu method was used to determine the total phenolic content. The test samples (1 mL) were mixed with sodium carbonate (2%, *w*/*v*) and the Folin-Ciocalteu phenol reagent (10%, *v*/*v*), and the mixture was allowed to stand for 10 min. The absorbance of the mixture was measured at 750 nm. The results were expressed as milligrams of gallic acid equivalents per gram of the sample [17].

### 3.6. Antibacterial Activity Assay

All of the strains tested were kindly donated by Prof. Jin-Cheol Yoo, Chosun University, Korea [21,22]. Vancomycin was used as a reference antibiotic to compare the antibacterial activity. The MIC values of the extract and reference antibiotic were determined by a conventional agar dilution method, as previously reported [21].

### 3.7. Statistical Analysis

A *p*-value of less than 0.05 was considered statistically significant using a Student’s *t*-test between two means for unpaired data or a Tukey’s HSD test posteriori analysis of variance (ANOVA) among three means for unpaired data.

## 4. Conclusions

In the present study, various solvent extracts of the QA leaf were successfully prepared and their chemical profiles and biological activities were evaluated. The methanolic extract showed the highest DPPH radical scavenging activity and total phenolic content, while the reducing power was the highest in the water extract. The ethyl acetate extract showed the highest antibacterial activity against *S. aureus* and also exerted antibacterial activity against *S. aureus* KCTC1928, *M. luteus* ATCC 9341, *S. typhimurium* KCTC 1925, *E. coli* KCTC 1923, and eight MRSA strains. The extracts and the analyzed active substances that were identified in this study were closely associated with antioxidant and antibacterial activities. Thus, the methanol and ethyl acetate extracts of QA have the potential to be applied therapeutically to various forms of antioxidant imbalance and infectious diseases that are caused by *S. aureus*. To the best of our knowledge, this is the first report on the antioxidant and antibacterial activity of various extracts from the QA leaf and active constituents therein. However, further investigation is required to confirm the pharmacological potentials of the extracts and to assess their safety. These efforts could lead to the development of the QA leaf as a promising, effective antioxidant and anti-infective agent.

## Figures and Tables

**Table 1:** Identified substances from the *Quercus acuta* (QA) extracts.

RT (min)	Compound	Quality	M.W	H (%)	EA (%)	A (%)	Et (%)	Me (%)	W (%)
26.089	Cinnamic acid	99	308.126	0	0.7	0.25	0	0	0
27.136	Hexadecanoic acid	99	328.28	4.91	6.19	5.15	5	0.69	0
28.309	Phytol	99	368.347	2.53	3.72	3.09	0	0	0
28.658	9,12-Octadecadienoic acid	99	352.28	1.38	1.37	1.14	0	0	0
28.716	α-Linolenic acid	99	350.264	3.24	3.6	2.73	0	0	0
34.077	Tetracosane	96	338.391	2.77	1.08	0.51	0	0	0
36.2	α-tocopherol	99	502.421	7.27	5.29	4.37	0	0	0
38.752	β-sitosterol	99	486.426	7.83	8.08	6.58	14.32	0.79	0
39.158	β-amyrin	99	498.426	10.69	11.74	9.55	6.52	0	0
39.891	2-Furancarboximidic acid	91	312.075	17.46	13.04	10.55	0	0	0
42.38	Friedelan-3-one	98	426.386	3.72	2.07	3.13	5.23	0	0

H: hexane extract, EA: ethyl acetate extract, A: acetone extract, Et: ethanol extract, Me: methanol extract, W: water extract.

**Table 2:** Contents (mg/g) of (+)-catechin, (-)-epicatechin, and taxifolin from the QA extracts (*n* = 5).

Extract	Extraction Yield (%)	(+)-Catechin	(-)-Epicatechin	Taxifolin
H	0.65	-	-	-
EA	0.98	7.32 ± 0.47	1.51 ± 0.01	0.95 ± 0.02
Ace	1.54	14.15 ± 0.09	2.05 ± 0.01	2.53 ± 0.1
MeOH	11.96	27.04 ± 0.48	3.05 ± 0.03	2.56 ± 0.05
EtOH	13.68	19.25 ± 0.49	2.27 ± 0.14	2.40 ± 0.39
Water	12.01	15.71 ± 0.29	3.43 ± 0.12	1.53 ± 0.09

**Table 3:** Antioxidant activity of QA extracts (*n* = 5).

Extract	DPPH Scavenging Activity IC_50_ (μg/mL)
Vitamin C (control)	8.18 ± 0.28
H	1008.23 ± 56.33
EA	438.37 ± 72.49
Ace	149.63 ± 22.11
MeOH	49.58 ± 1.46
EtOH	59.01 ± 6.44
Water	73.67 ± 3.08

**Table 4:** Reducing power and total phenolic content of the QA extracts (*n* = 5).

Extract	Reducing Power (Ascorbic Acid eq. μg/100 μg Extract)	Total Phenolic Content (Gallic Acid eq. mg/g)
H ex	4.73 ± 0.04	1.53 ± 0.05
EA ex	28.52 ± 0.29	10.37 ± 0.18
Ace ex	61.00 ± 0.47	21.38 ± 0.51
MeOH	163.69 ± 1.37	83.25 ± 2.39
EtOH	151.39 ± 2.42	85.20 ± 0.89
Water	171.57 ± 0.93	74.21 ± 1.04

**Table 5:** Antibacterial activity of the ethyl acetate extracts from QA leaf.

Organisms	MIC(μg/mL)	
	Extract	Vancomycin
*Alacligenes faecalis* ATCC 1004	>1000	>80
*Enterococcus Faecalis* ATCC 29212	>1000	1.25
*Bacillus subtilis* ATCC6633	>1000	0.625
*Staphylococcus aureus* KCTC 1928	125	1.25
*Micrococcus luteus* ATCC 9341	500	1.25
*Mycrobacterium smegmatis* ATCC 9341	>1000	2.5
*Salmonella typhimrium* KCTC 1925	250	>80
*Escherrichia coli* KCTC 1923	250	>80
*Pseudomonas aeruginosa* KCTC	>1000	>80
MRSA 693E	125	1.25
MRSA 4-5	250	>80
MRSA 5-3	125	>80
VRE 82	>1000	>80
VRE 89	>1000	>80
VRE 98	>1000	>80
VRSA(MRSA2-32)	>1000	>80
MRSA S1	125	2.5
MRSA S3	250	1.25
MRSA U4	125	0.625
MRSA P8	125	1.25
MRSA B15	250	1.25
IMP 100	>1000	>80
IMP 102	>1000	>80
IMP 120	>1000	>80
IMP 123	>1000	>80
IMP 129	>1000	>80
VRE 2	>1000	>80
VRE 3	>1000	>80
VRE 4	>1000	>80
VRE 5	>1000	>80
VRE 6	>1000	>80
ESBL LMH-B1	>1000	>80
ESBL LMH-P3	>1000	>80
ESBL LMH-S1	>1000	>80
ESBL LMH-U4	>1000	>80

MRSA: methicillin-resistant *S. aureus*, VRSA: vancomycin-resistant *S. aureus* (VRSA), VRE: vancomycin-resistant enterococci, IMP: carbapenemase producing *P. aeruginosa*, ESBL: extended spectrum β-lactamase producing *E. coli*.

**Table 6:** Analytical conditions of high-performance liquid chromatography (HPLC) system to analyze the three markers.

Parameters	Conditions		
Column	Zorbax extended-C18 (C18, 4.6 mm × 150 mm, 5 µm)		
Flow rate	0.8 mL/min		
Injection volumn	10 μL		
UV detection	230 nm		
Run time	35 min		
Gradient	**Time (min)**	**A(%)**	**B(%)**
	0	10	90
	10	10	90
	20	20	80
	25	30	70
	27	100	0
	28	10	90
	35	10	90
